# High genomic variability in the plant pathogenic bacterium *Pectobacterium parmentieri* deciphered from de novo assembled complete genomes

**DOI:** 10.1186/s12864-018-5140-9

**Published:** 2018-10-16

**Authors:** S. Zoledowska, A. Motyka-Pomagruk, W. Sledz, A. Mengoni, E. Lojkowska

**Affiliations:** 1 Department of Biotechnology, Intercollegiate Faculty of Biotechnology, University of Gdansk and Medical University of Gdansk, Gdansk, Poland; 2 Department of Biology, University of Florence, Sesto Fiorentino, Florence, Italy

**Keywords:** Blackleg, Pectinolytic erwinias, Pan-genome, Comparative genomics, Phages, Bacterial evolution, Genome plasticity

## Abstract

**Background:**

*Pectobacterium parmentieri* is a newly established species within the plant pathogenic family *Pectobacteriaceae*. Bacteria belonging to this species are causative agents of diseases in economically important crops (e.g. potato) in a wide range of different environmental conditions, encountered in Europe, North America, Africa, and New Zealand. Severe disease symptoms result from the activity of *P. parmentieri* virulence factors, such as plant cell wall degrading enzymes. Interestingly, we observe significant phenotypic differences among *P. parmentieri* isolates regarding virulence factors production and the abilities to macerate plants. To establish the possible genomic basis of these differences, we sequenced 12 genomes of *P. parmentieri* strains (10 isolated in Poland, 2 in Belgium) with the combined use of Illumina and PacBio approaches. De novo genome assembly was performed with the use of SPAdes software, while annotation was conducted by NCBI Prokaryotic Genome Annotation Pipeline.

**Results:**

The pan-genome study was performed on 15 genomes (12 de novo assembled and three reference strains: *P. parmentieri* CFBP 8475^T^, *P. parmentieri* SCC3193, *P. parmentieri* WPP163). The pan-genome includes 3706 core genes, a high number of accessory (1468) genes, and numerous unique (1847) genes. We identified the presence of well-known genes encoding virulence factors in the core genome fraction, but some of them were located in the dispensable genome. A significant fraction of horizontally transferred genes, virulence-related gene duplications, as well as different CRISPR arrays were found, which can explain the observed phenotypic differences. Finally, we found also, for the first time, the presence of a plasmid in one of the tested *P. parmentieri* strains isolated in Poland.

**Conclusions:**

We can hypothesize that a large number of the genes in the dispensable genome and significant genomic variation among *P. parmentieri* strains could be the basis of the potential wide host range and widespread diffusion of *P. parmentieri*. The obtained data on the structure and gene content of *P. parmentieri* strains enabled us to speculate on the importance of high genomic plasticity for *P. parmentieri* adaptation to different environments.

**Electronic supplementary material:**

The online version of this article (10.1186/s12864-018-5140-9) contains supplementary material, which is available to authorized users.

## Background

Genomes of a given bacterial species can show considerable variation in gene content, distribution, and presence of mobile elements. Therefore, conducting systematic analyses of the entire gene repertoire of several strains from a specific species, termed the pan-genome study, is vital for understanding bacterial intraspecies diversity, population genetics, and bacterial evolution [1]. The core pan-genome constitutes of highly-conserved genes present in all the analyzed genomes and usually encodes genes related to fundamental aspects of the bacterial biology contributing to significant phenotypic traits [2]. It has been reported previously that some genes of the dispensable (accessory and unique) pan-genome might play a role in bacterial adaptation to specific growth conditions, such as colonization of new ecological niches, symbiosis, host-cell interaction, and pathogenicity. In other words, the plasticity of this pan-genome fraction contributes to bacterial evolution [3]. The diversity/plasticity mentioned above is especially crucial regarding opportunistic bacterial pathogens, such as human, animal and also plant pathogens spreading in new hosts and/or a new environment.

Specifically, the genomes of a plant quarantine pathogen *Ralstonia solanacearum* include chromosomal rearrangements and several genes recently acquired via a horizontal gene transfer (HGT) [4–6]. *R. solanacearum* strains exhibit an unusually broad host range as they can infect more than 250 plant species in monocot and dicot botanical families [7]. The vast host range of *R. solanacearum* may be correlated with its high genomic plasticity [8–10] attributed to the occurrence of Mobile Genetic Elements (MGE), such as phages and plasmids [11].

Amongst plant pathogenic bacteria that trigger economically important losses, the causative agents of soft rot and potato blackleg should be listed. This subgroup of Gram-negative *Gammaproteobacteria,* classified to the *Pectobacteriaceae* family currently encloses two genera: *Dickeya* and *Pectobacterium* [12]. The diseases caused by *Dickeya* spp. and *Pectobacterium* spp. result from the activity of Plant Cell Wall Degrading Enzymes (PCWDE) such as pectinases, cellulases, and proteases secreted via Type I or II secretion systems [13]. The Broad host range of these phytopathogens can be exemplified by the fact that *Pectobacteriaceae* have been reported to cause soft-rotting symptoms in a large number of plants, including 16 dicot plant families in 11 orders and 11 monocot families in 6 orders [14].

The development of novel diagnostic methods resulted in several reclassifications within both genera. High genomic heterogeneity was attributed to *Pectobacterium carotovorum* strains (exhibiting about 20 different *recA* PCR-RFLP patterns) in comparison to *Pectobacterium atrosepticum* (with just two *recA* PCR-RFLP patterns [15]). The application of molecular techniques: genomic sequences comparison, DNA:DNA hybridization and average nucleotide identity (ANI) analysis resulted in the reevaluation of the taxonomic position of *P. carotovorum* strains deposited in different collections and/or isolated in the recent years. Finally, some strains of *P. carotovorum*, including the most frequently studied *P. carotovorum* SCC3193, were reclassified to *Pectobacterium wasabiae* [16] and later on to *Pectobacterium parmentieri* species [17]*.*

In this study, we focused on *P. parmentieri* infecting plants worldwide and detected in Europe, North America, Africa, Asia and New Zealand [17–25]. *P. parmentieri* has been proven to cause disease symptoms on potato plants and tubers and also to survive in unfavourable environmental conditions (such as soil or surface waters). By now, their host range in the natural environment has not been fully understood. However, there is some information about the isolation of *P. parmentieri* (there *P. wasabiae*) strains from cabbage, eggplants, sweet potato and tomato in Malaysia [26].

Currently, there are five publicly available genomes of *P. parmentieri* (NCBI servers, June 2018)*,* whereas only 3 of them are closed to a full chromosome. The genome (GenBank accession: NZ_CP015749.1) of the type strain *P. parmentieri* CFBP 8475^T^ (previously *P. wasabiae* RNS 08.42.1A) is 5.03 Mb in size with GC content of 50.4% [27]. Among the 4462 proteins it encodes, there have been described some proteins speculated to ensure specific phenotypic traits that are important for *P. parmentieri* virulence and adaptation to its primary host - potato, such as PCWDE, components of secretion systems and AHL-dependent quorum sensing system [27].

The aim of the here presented study is to elucidate the genomic basis of *P. parmentieri* spread and rapid adaptation to different climate conditions (temperature, humidity). It was achieved by completing genome sequencing of 12 *P. parmentieri* strains isolated from potato plants in various environments, followed by comparative genomic analyses conducted on these twelve strains and three other sequences available in GenBank. The obtained results point to a high abundance of MGE characterizing the reported *P. parmentieri* pan-genome, which may likely be linked with adaptation to different environmental niches and be the reason for the worldwide spreading of this species.

## Results and discussion

### Phenotypic characterization of *P. parmentieri* strains

Phenotypic characterization of the available *P. parmentieri* strains, isolated in Poland (10), Belgium (2), Finland (1) and France (1) (Table 1), was performed. We observed high variability between the analyzed strains regarding the potato maceration efficacy. It was interesting that the lowest and the highest maceration ability was noted for the strains isolated in Poland. Besides, IFB5408 indicates 4-fold lower maceration than IFB5626 (Table 2).

**Table 1:** Strains used in this study

Strain number	Species	Year and country of isolation	Host	Accession number	Reference
IFB5408	*P. parmentieri*	2013, Poland	Potato stem	CP026977	[27]
IFB5427	*P. parmentieri*	2013, Poland	Weed	CP027260	[27]
IFB5432	*P. parmentieri*	2013, Poland	Potato tuber	CP026979	[27]
IFB5441	*P. parmentieri*	2013, Poland	Potato tuber	CP026980	[27]
IFB5485, GBBC 1786	*P. parmentieri*	2012, Belgium	Potato	CP026981	This study
IFB5486, GBBC 1809	*P. parmentieri*	2012, Belgium	Potato	CP026982	This study
IFB5597	*P. parmentieri*	2014, Poland	Potato stem	PSZH00000000	[27]
IFB5604	*P. parmentieri*	2014, Poland	Potato stem	CP026983	[27]
IFB5605	*P. parmentieri*	2014, Poland	Potato stem	CP026984	[27]
IFB5619	*P. parmentieri*	2014, Poland	Potato stem	CP026985	[27]
IFB5623	*P. parmentieri*	2014, Poland	Potato stem	CP026986	[27]
IFB5626	*P. parmentieri*	2014, Poland	Potato tuber	PSZG00000000	[27]
CFBP 8475^T^	*P. parmentieri*	2008, France	Potato	NZ_CP015749.1	[36]
SCC3193	*P. parmentieri*	1980s, Finland	Potato	NC_017845.1	[39]
WPP0163	*P. parmentieri*	2004, USA	Potato	NC_013421.1	[23]

**Table 2:** Comparison of the maceration ability and phenotypic features: enzyme activities, siderophores production, motility and biofilm formation of the tested *P. parmentieri* strains

Strains	Maceration, mm	Pectinases, mm	Cellulases, mm	Proteases, mm	Lipases, mm	Siderophores, mm	Swimming, mm	Swarming, mm	Biofilm, OD525
IFB5408	9.5 ± 1.93	20.7 ± 0.46	10.3 ± 0.30	6.8 ± 0.20	11.1 ± 0.37	13.8 ± 0.50	42.6 ± 2.94	11.3 ± 0.83	0.113 ± 0.0017
IFB5427	31.0 ± 0.86	21.4 ± 0.57	10.9 ± 0.31	10.5 ± 0.19	10.6 ± 0.28	14.3 ± 0.56	10 ± 1.02	11.7 ± 0.74	0.094 ± 0.0021
IFB5432	15.0 ± 0.96	22.2 ± 0.50	9.8 ± 0.29	12.2 ± 0.49	11.7 ± 0.52	8.7 ± 0.49	19.4 ± 4.22	8.4 ± 0.14	0.062 ± 0.0055
IFB5441	11.7 ± 1.44	21.0 ± 0.42	9.8 ± 0.30	11.1 ± 0.31	11.3 ± 0.27	7.67 ± 0.18	11.7 ± 1.38	8.4 ± 0.26	0.064 ± 0.0091
IFB5485	27.4 ± 2.03	20.9 ± 0.26	11.8 ± 0.25	10.9 ± 0.19	11.3 ± 0.35	12.8 ± 0.36	19.7 ± 2.78	21.3 ± 0.49	0.080 ± 0.0042
IFB5486	29.3 ± 0.81	20.5 ± 0.39	11.8 ± 0.65	6.6 ± 0.14	10.3 ± 0.27	14.6 ± 0.26	8.5 ± 0.71	18.8 ± 0.70	0.084 ± 0.0064
IFB5597	12.1 ± 0.92	25.3 ± 0.31	7.1 ± 0.31	13.2 ± 0.56	11.1 ± 0.54	6.3 ± 0.37	32.5 ± 2.51	13.6 ± 1.53	0.079 ± 0.0028
IFB5604	14.4 ± 0.84	25.6 ± 0.28	0.00	10.8 ± 0.42	11.0 ± 0.21	17.7 ± 0.35	25.3 ± 1.09	9.9 ± 0.65	0.108 ± 0.0063
IFB5605	20.9 ± 0.87	24.5 ± 0.50	0.00	0.00	10.8 ± 0.32	14.7 ± 0.51	36.5 ± 5.34	9.9 ± 0.35	0.098 ± 0.0058
IFB5619	25.7 ± 0.61	26.8 ± 0.30	8.2 ± 0.32	0.00	11.0 ± 0.30	13.6 ± 0.78	9.6 ± 0.76	28.6 ± 1.04	0.093 ± 0.0085
IFB5623	13.6 ± 0.89	25.1 ± 0.28	0.00	10.5 ± 0.48	10.7 ± 0.31	8.6 ± 0.22	59.4 ± 3.00	26.6 ± 0.86	0.068 ± 0.0057
IFB5626	38.2 ± 1.20	24.7 ± 0.48	0.00	0.00	12.0 ± 0.27	16.6 ± 0.39	15.6 ± 2.73	25.8 ± 1.18	0.072 ± 0.0073
CFBP 8475^T^	34.3 ± 0.64	23.1 ± 0.35	11.3 ± 0.21	10.3 ± 0.18	11.1 ± 0.41	8.2 ± 0.16	14.8 ± 1.87	21.1 ± 1.41	0.089 ± 0.0072
SCC3193	11.2 ± 1.21	23.2 ± 0.83	6.7 ± 0.31	0.00	10.8 ± 0.47	0.00	22.6 ± 0.98	8.1 ± 0.26	0.063 ± 0.0108
*P. aeruginosa* PA01	nt.	nt.	nt.	nt.	nt.	nt.	nt.	nt.	0.113 ± 0.0030
*Significance code*	***	**	**	**	*No differences*	**	***	***	**

The values represent averages from three biological repetitions and standard error

nt - not tested;

Significance codes: 0 ‘***’ 0.001 ‘**’ 0.01 ‘*’ generated form ANOVA analysis indicating the statistical differences within the analyzed phenotypic feature

The investigation of other *P. parmentieri* phenotypic features, such as the activity of PCDWEs revealed statistically significant differences between strains. The tested strains indicated significant differences in pectinases, cellulases and proteases but not lipases activities (Table 2). Strains IFB5597, IFB5604, IFB5619, IFB5623 produced significantly higher amounts of pectinases. At the same time, none of them produced high levels of cellulases. However, IFB5597 as the only one of them indicate high activity of proteases. On the other hand strains IFB5408, IFB5441, IFB5485 and IFB5486 possessed the low activity of pectinases, but IFB5485 and IFB5486 produced significant amounts of cellulases.

The studied *P. parmentieri* strains exhibit significant differences regarding both swimming and swarming motility (Table 2). Conserning biofilm formation, IFB5408, IFB5604 and IFB5605 isolates were as efficient in this trait as *P. aeruginosa* PAO1, a strain commonly applied as a positive control in biofilm formation assessment (Table 2).

Furthermore, we calculated correlation coefficients between different phenotypic traits by applying Pearson statistical method (*p* < 0.05). Weak positive linear relationships between the strain’s ability to macerate potato tissue and their cellulases activity in addition to biofilm formation capacity (0.118 and 0.085) were observed. Strains possessing the overall moderate activity of pectinases, cellulases and proteases showed the highest ability to macerate potato tissue. In other words, the observed effects of different PCDWEs are synergistic, and the individual activity of each enzyme tends to complement each other (Table 2).

The here-described *P. parmentieri* intraspecies variation is in agreement with several previous studies, in which *P. parmentieri* isolates differed in the plant maceration efficacy and PCWDE activities [20, 21, 23, 24, 28].

### Genome structure of the *P. parmentieri* strains

Comparative genomics on 15 *P. parmentieri* strains (12 newly sequenced and three available from the GenBank) was performed. The number of scaffolds, GC content and the amount of N bases for all of the analyzed genomes were determined (Table 3). Ten of the newly assembled genomes were closed to a full chromosome, but in two cases we were not able to close specific genomes: strain IFB5626 consists of 2, and IFB5597 of 5 scaffolds. The length of the obtained *P. parmentieri* genomes varied from 4,877,201 bp (IFB5604) to 5,125,304 bp (IFB5427), which equals approx. 5% difference. GC content varied between 50.29 and 50.6%, where the strain IFB5441 and IFB5619 had the lowest and the highest values, respectively. These results are in agreement with those obtained earlier for *P. parmentieri* CFBP 8475^T^ and *P. parmentieri* SCC3193 regarding the genome length (5,030,841 bp and 5,164,411 bp) and the GC content (50.4% and 50.37%) [16, 17].

**Table 3:** Detailed pan-genome statistics and shape

Strain	GenBank Accession no.	Genome size	No. of N bases^a^	No of scaffolds	GC%	Total number of genes	Genes encoding					Pan-genome^b^		
							Proteins	rRNAs	tRNAs	ncRNA	CRISPR	Core genes	Accessory genes	Unique genes
*P. parmentieri* strains														
IFB5408	CP026977	5,022,017	0	1	50.40	4707	4420	22	77	8	4	3706	756	45
IFB5427	CP027260	5,125,304	0	1	50.41	4842	4561	22	76	8	4	3706	808	135
IFB5432	CP026979	5,010,533	0	1	50.45	4709	4406	22	77	8	4	3706	632	133
IFB5441	CP026980	5,082,177	0	1	50.29	4782	4492	22	76	9	4	3706	698	177
IFB5485	CP026981	4,885,249	0	1	50.56	4534	4252	22	77	8	4	3706	619	7
IFB5486	CP026982	5,038,122	0	1	50.49	4783	4454	22	77	8	4	3706	822	12
IFB5597	PSZH00000000	5,046,647	0	5	50.57	4734	4443	22	77	7	4	3706	652	174
IFB5604	CP026983	4,877,201	0	1	50.56	4535	4249	22	77	8	4	3706	610	19
IFB5605	CP026984	5,040,694	100	1	50.44	4762	4494	22	77	8	4	3706	811	58
IFB5619	CP026985	4,959,302	0	1	50.60	4612	4328	22	77	8	4	3706	673	31
IFB5623	CP026986	5,006,798	100	1	50.33	4713	4422	22	77	8	4	3706	694	112
IFB5626	PSZG00000000	5,059,731	100	2	50.48	4790	4519	22	77	8	4	3706	810	85
CFBP 8475^T^	NZ_CP015749.1	5,030,841	0	1	50.40	4772	4462	22	77	8	4	3706	755	40
SCC3193	NC_017845.1	5,164,411	0	1	50.37	4841	4449	22	78	8	4	3706	486	346
WPP0163	NC_013421.1	5,063,892	0	1	50.48	4690	4423	22	75	8	4	3706	604	181
*P. parmentieri* plasmid *pPAR01*														
pPAR01		CP027260	101,998	0	1	49.45								

^a^Number of undetermined bases

^b^The pan-genome content has been calculated on PGAP annotated genomes

**Fig. 1:**
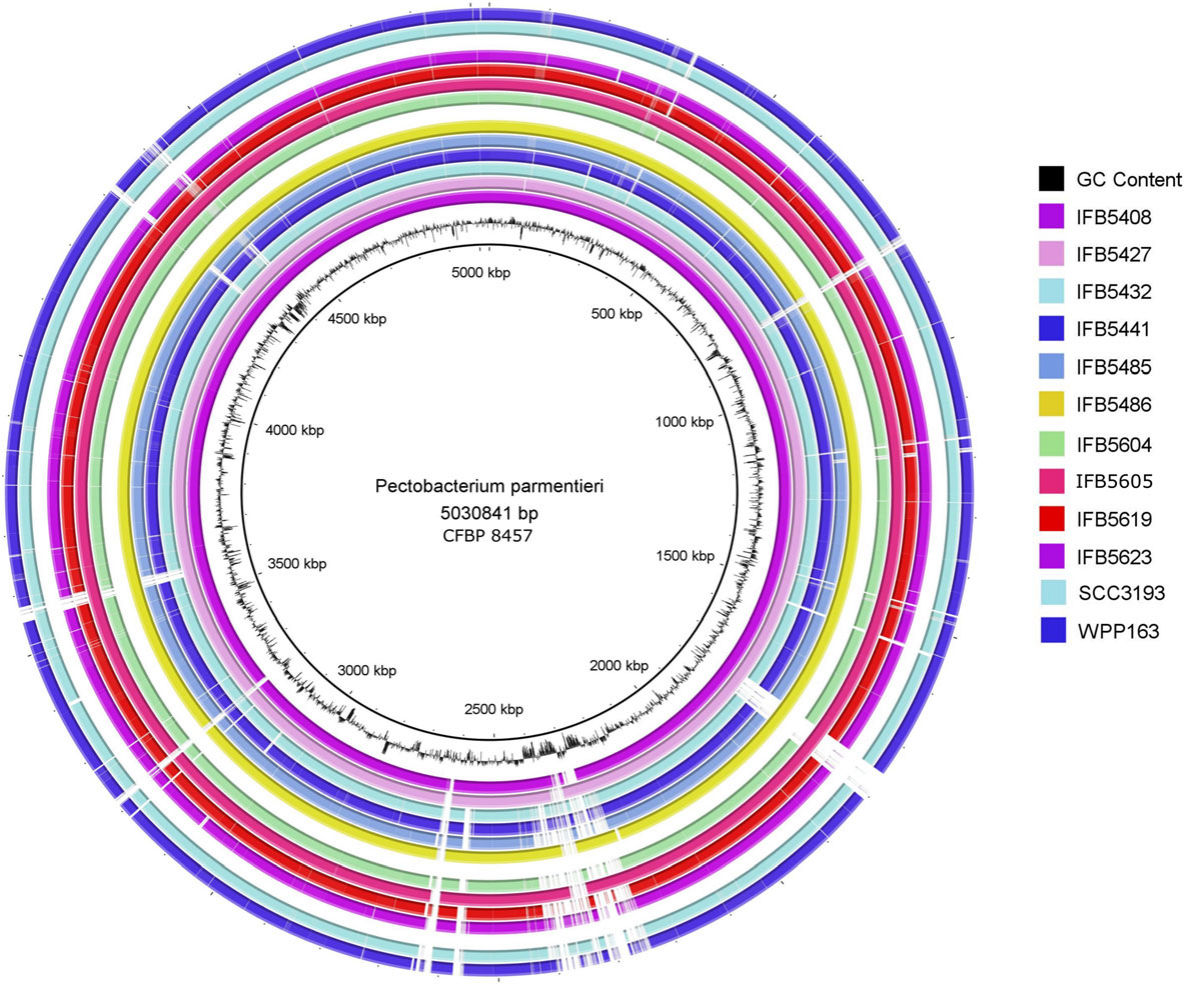
*P. parmentieri* genomes shape generated with the use of BRIG software

Obtaining complete genome sequences allowed us to investigate deeply structural variations within the *P. parmentieri* genomes. The synteny of the closed genomes is presented in Fig. 1. In general, genomes were highly syntenic with minor structural differences. We detected a sizeable chromosomal rearrangement in one of the strains that have been isolated in Belgium (IFB5486). This deviation might have appeared during the long-time period of evolution, which could have subsequently undergone selective pressure under specific environmental conditions during the lengthy process of speciation, as it was reported before in other members of *Enterobacteriales* [29]. Moreover, highly variable regions in almost all of the analyzed strains were detected (Fig. 1). In these highly variable regions, genes encoding phage-related proteins were found (Fig. 1) and a more precise description of these MGE shall be provided later.

Interestingly, during de novo genome assembly of IFB5427 strain, the presence of a large plasmid of about 100 kb has been discovered. This plasmid, for the first time described in *P. parmentieri* species, was named *pPAR01*. Regarding the *Pectobacteriaceae* genus, the presence of other plasmids was reported only in *P. carotovorum* SCC1 (5 kb), *D. solani* PPO9091 (43 kb) and *D. fangzhongdai* DSM 101947 (5 kb) [30–32]*.* Notably, the above-listed plasmids are much smaller than *pPAR01*. A more detailed analysis of this plasmid is presented in ‘the mobilome’ paragraph below.

### *P. parmentieri* has an open pan-genome

With the NCBI Prokaryotic Genome Annotation Pipeline, the number of total predicted genes varied from 4842 to 4534 for strains IFB5427 and IFB5485, respectively. The total number of genes encoding proteins ranged from 4561 to 4252 (Table 3) and was attributed to the strains mentioned above.

**Fig. 2:**
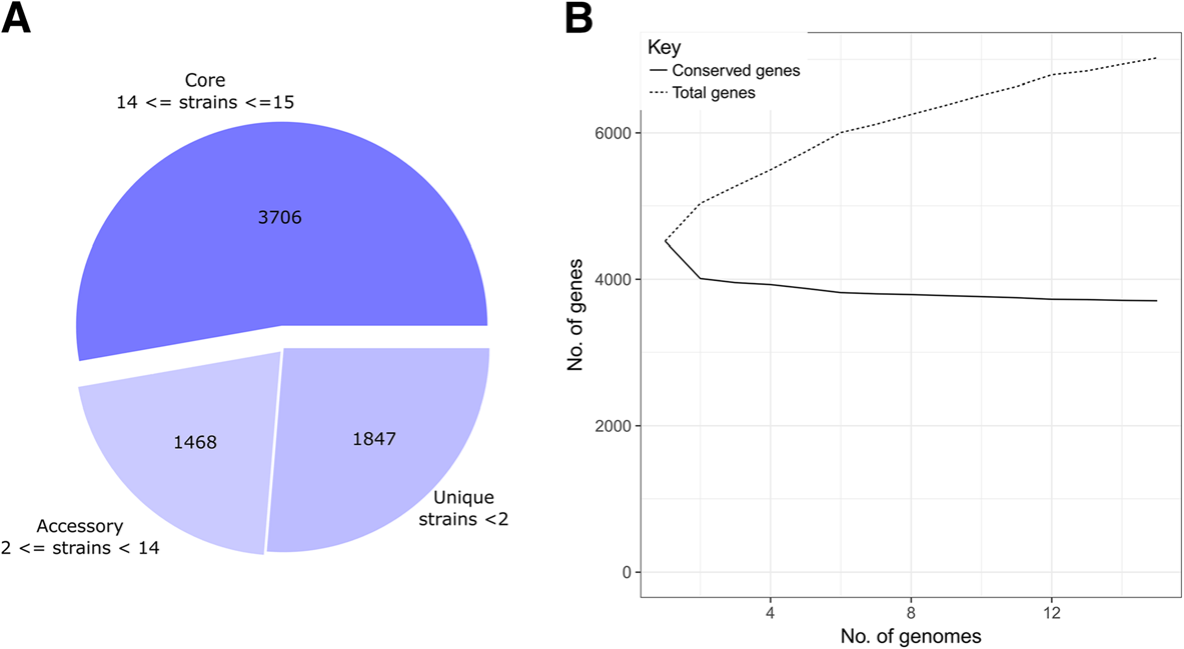
The pan-genome shape of *P. parmentieri.* **a** The pan-genome pie chart showing gene content visualised with the use of Roary software. **b** Heap’s law chart representation regarding conserved genes vs total genes in 15 genomes

The pan-genome shape of the 15 analyzed *P. parmentieri* genomes is presented in Fig. 2A. A total of 7021 gene clusters (orthologs) were found, 3706 of which comprised the core genome (52.8%), 1468 the accessory genome (20.9%) and 1847 (26.3%) the unique genome fraction (Fig. 2a, Table 3). The quantity of the determined accessory genes varied from 486 to 822 for the most diverse strains SCC3193 and IFB5486, respectively. Interestingly, *P. parmentieri* SCC3193 indicated the highest quantity of unique genes (346 genes), while the strain IFB5485 the lowest (7 genes).

To investigate the openness of *P. parmentieri* pan-genome Heap’s Law was utilized [33, 34]. The openness of pan-genome reflects the diversity of the gene pool within bacterial species. For species with an open pan-genome, an addition of a newly sequenced genome significantly changes the pan-genome size [2], contrarily to what is observed in the case of strains exhibiting a closed pan-genome. The calculated alpha value of 0.81 indicated that the *P. parmentieri* pan-genome is open (Fig. 2b) and that could be the result of high genomic plasticity within this species [1]. This finding is in agreement with the level of openness of the pan-genome for other representatives of *Gammaproteobacteria* as it was reported before [35–38]. In contrast to *P. parmentieri* pan-genome, closely related and very homogenous *D. solani* species have a closed pan-genome [39].

### Genomic relatedness of *P. parmentieri* strains

**Fig. 3:**
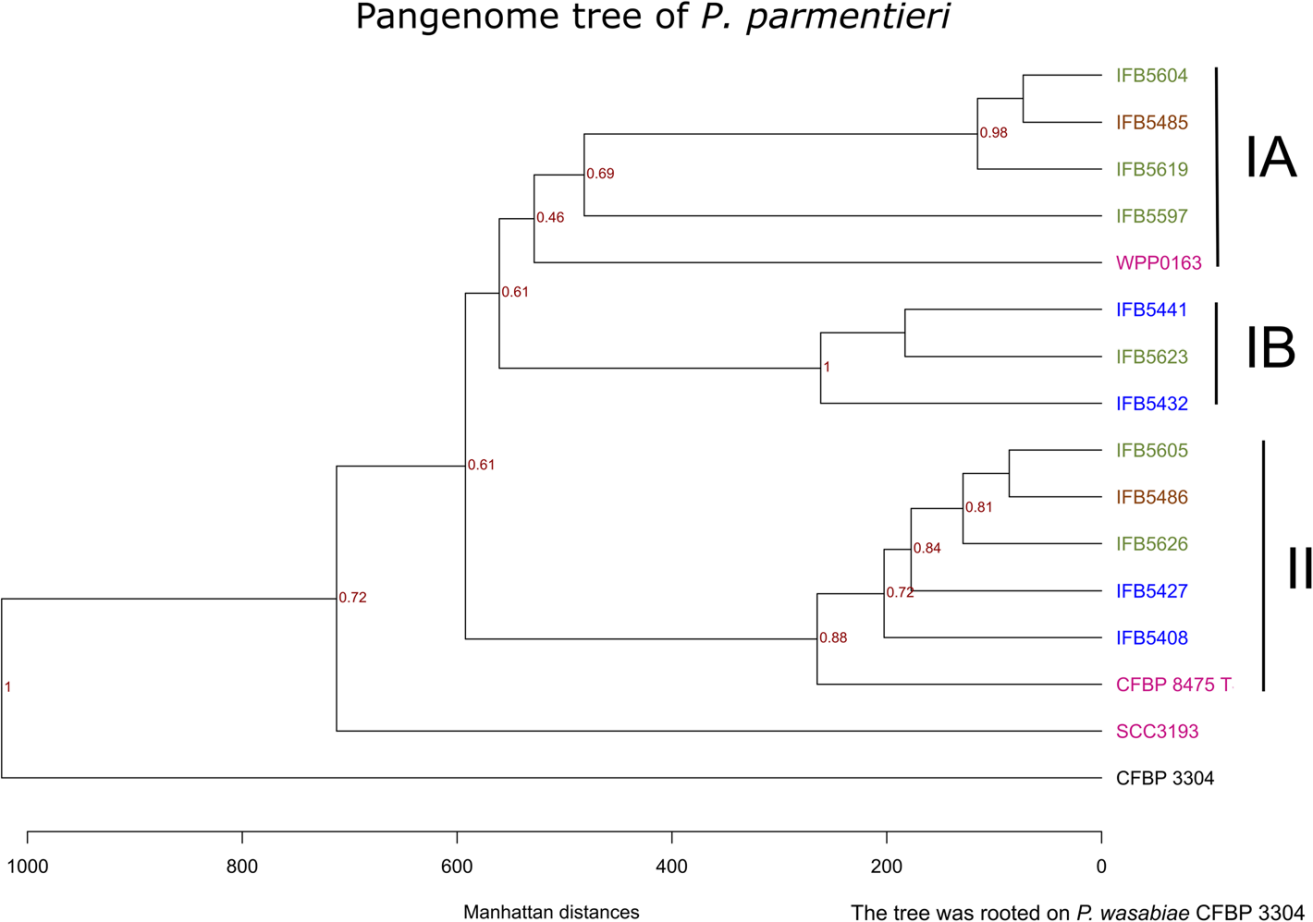
Phylogenetic tree constructed with the method blast all against all using protein sequences. The tree was rooted on *P. wasabiae* CFBP 3304. The color codes: blue – strains isolated in 2013 (Poland), green – strains isolated in 2014 (Poland), brown – strains isolated in Belgium, pink – reference strains

Genetic relatedness among the *P. parmentieri* strains was studied by both computing the ANI values and by establishing genomic similarities with a pan-genome based analysis. As expected for strains belonging to the same species, high ANI values ranging from 98.98 to 99.97 were observed. Phylogenetic analyses derived from the comparison of all protein sequences encoded within the genomes is presented in Fig. 3. The genomes of *P. parmentieri* strains formed two main clades, from which the first (Clade I) is further divided into two subclades (IA and IB). Clade IA comprises genomes of *P. parmentieri* reference strain WPP0163 (isolated in the USA), three strains isolated in Poland in the year 2014 (IFB5597, IFB5604, and IFB5619) and also one strain isolated in Belgium (IFB5485) (Fig. 3). Clade IB, on the other hand, encloses genomes of three strains isolated in Poland, two in the year 2013 (IFB5432, IFB5441) and one strain from 2014 (IFB5623). The second clade (Clade II) consists of genomes of *P. parmentieri* CFBP 8475^T^, two strains isolated in 2013 (IFB5408, IFB5427), two in 2014 in Poland (IFB5605, IFB5626) and one strain isolated in Belgium (IFB5486). Interestingly, *P. parmentieri* SCC3193 being the model strain used for studying *P. parmentieri* molecular biology for many years (previously *P. c.* subsp. *carotovorum* SCC3193/*P. wasabiae* SCC3193 [40–42]) is distinctively separated from the clades mentioned above (has the highest number of unique genes). Furthermore, strains constituting Clade II, share the highest quantity of accessory genes (on average 794) in contrast to strains grouped within Clade I, for which an average number of accessory genes is 645) (Fig. 3, Table 3). The above-presented grouping corresponds with the ANI-based clustering as ANI Cluster I reflects Clade II, while ANI Cluster II is consistent with Clade IB (Additional file 1: Figure S1A).

In general, *P. parmentieri* strains did not group accordingly to geographical site of isolation (Fig. 3), which may suggest their high geographical mobility. However, to fully understand the biogeographical pattern of genomic variation in *P. parmentieri*, a more significant number of strains from different regions should be sequenced and analyzed.

The described phylogenetic pattern was further supported by inspection of the presence/absence of genes in the dispensable (accessory and unique) genome fraction (Additional file 1: Figure S1B), which also showed agreement with the groupings presented in the phylogenetic analysis of all protein sequences (Fig. 3). We observe that all the strains from Clade II (Fig. 3) form a monophyletic group in Additional file 1: Figure S1B. Interestingly, *P. parmentieri* SCC3193 (distinctively separated from other strains in the protein phylogenetic tree), in accessory genome fraction phylogenesis, is grouping with *P. parmentieri* WPP163 and IFB5597.

### Functional annotation of the dispensable genome fraction

**Fig. 4:**
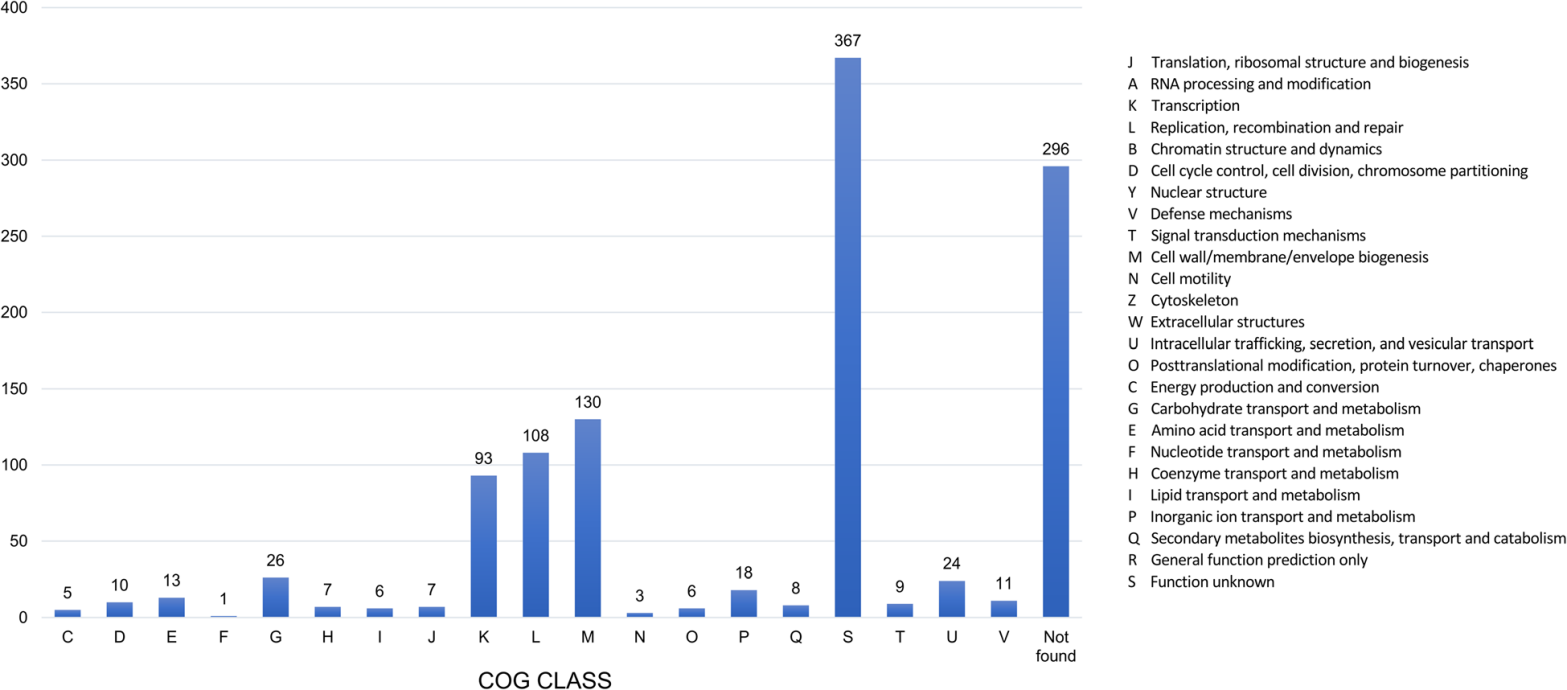
COG classes annotation of the accessory pan-genome fraction

To describe the strain-specific genomic differences, which are usually represented within the dispensable pan-genome fraction, we performed: class of genes (COG) annotation. It was done for every protein belonging to the dispensable pan-genome fraction to obtain results biased towards genes found in all the analyzed genomes (Fig. 4). We successfully annotated 900 protein sequences. A significant fraction of the proteins was attributed to cell wall and membranes biogenesis (130 genes) and replication and recombination (108 genes). In particular, among these 108 genes, there were quite a few proteins related to MGE, such as transposases, integrases and also DNA repair proteins and the CRISPR-associated ones.

Proteins related to transcription were also abundant (93 genes), such as LysR family transcriptional regulators, controlling numerous genes involved in virulence, metabolism, quorum sensing and motility [43], but also other regulators responsible for plasmid maintenance (Xre), initiation of expression of phage-related genes (Ogr/delta) or resistance to multiple antibiotics (MarR) [44]. Also, 26 proteins were found to be involved in carbohydrate transport and metabolism with the notable presence of pectate lyase, glucosidases, transporters or outer membrane porins (Additional file 2: Table S1). Almost the same number of proteins (24) were attributed to intracellular trafficking, secretion, and vesicular transport, including conjugal transfer proteins, filamentous hemagglutinin, Flp/Tad pilus component, especially crucial for the establishment of virulence [45] in addition to transfer proteins. Moreover, 18 proteins were designated to inorganic ion transport and metabolism, and in this COG group, we found, among others, TonB-dependent siderophore receptors capable of binding siderophore-iron complexes with high specificity and carrying out active transport across the outer membrane [46].

Besides, we have discovered that a significant part of COG class K is related to phage-related transcription regulators and phage-related proteins. Prophages, as genetic elements, may account for a significant fraction of the bacterial genome and pass to the chromosome many genes in a single event of integration [47].

### The variability within the pathogenome of *P. parmentieri*

Genes encoding virulence factors, motility features, chemotaxis, secretion system and quorum sensing components were investigated to describe the possible genomic variation related to pathogenesis comprehensively. Focusing on PCWDE, we have assessed the presence of genes encoding: pectinases, polygalacturonases, cellulases, and proteases. In more detail, genes encoding nine pectate lyases (*pel1, pel2, pel3, pelA, pelL, pelW_1, pelW_2, pelX* and putative *pelC*)*,* and also three genes coding for pectin esterases (*pemA, pemB,* and *rhgT*) are noted in the core genome fraction (Additional file 2: Table S1).

Interestingly, only one IFB5486 strain having sizeable chromosomal rearrangement (Fig. 1) did not possess the gene coding for a pectin lyase (*pnl*). However, even with the lack of *pnl*, this strain is still able to effectively destroy plant tissue components (Table 2) probably by producing all other well-known pectate lyases, pectate disaccharides hydrolases and/or pectinesterases. These results are in agreement with the previously performed study on *Dickeya dadantii* 3937 where mutations in single genes related to pectinolytic activity did not influence the overall maceration ability [48].

Regarding other PCWDE, we found 4 genes encoding polygalacturonases: *pehA_1*, *pehA_2, rhiE,* and *pehX*, 3 genes encoding cellulases (*celV*, *celS, bcsZ*)*,* and 6 genes coding for proteases (*prtS, prtC, prlC, pepT, htpX_1,* and *pcp_1*) in the *P. parmentieri* core genome fraction (Additional file 2: Table S1). Additionally, we confirmed the presence of a protease-encoding gene (*prtB_2*) in all the genomes except *P. parmentieri* SCC3193 strain. Also, genes encoding proteins of type 1 and 2 secretion systems, important for export of proteases, cellulases and pectinases are present in the core genome.

Focusing on the genes related to oligogalacturonides degradation, we determined the occurrence of two genes encoding 2-dehydro-3-deoxygluconokinase: *kdgK_1* and *kdgK_2* in the core pan-genome. Besides, genes coding for other essential enzymes involved in oligogalacturonide degradation, namely: *kduD,* two types of the *kduI* gene (*kduI_2, kduI_1*)*, kdgT*, *kdsC*, *kdsD*, *kdgM_3* and *ogl* were proven to be present in all the tested genomes.

**Fig. 5:**
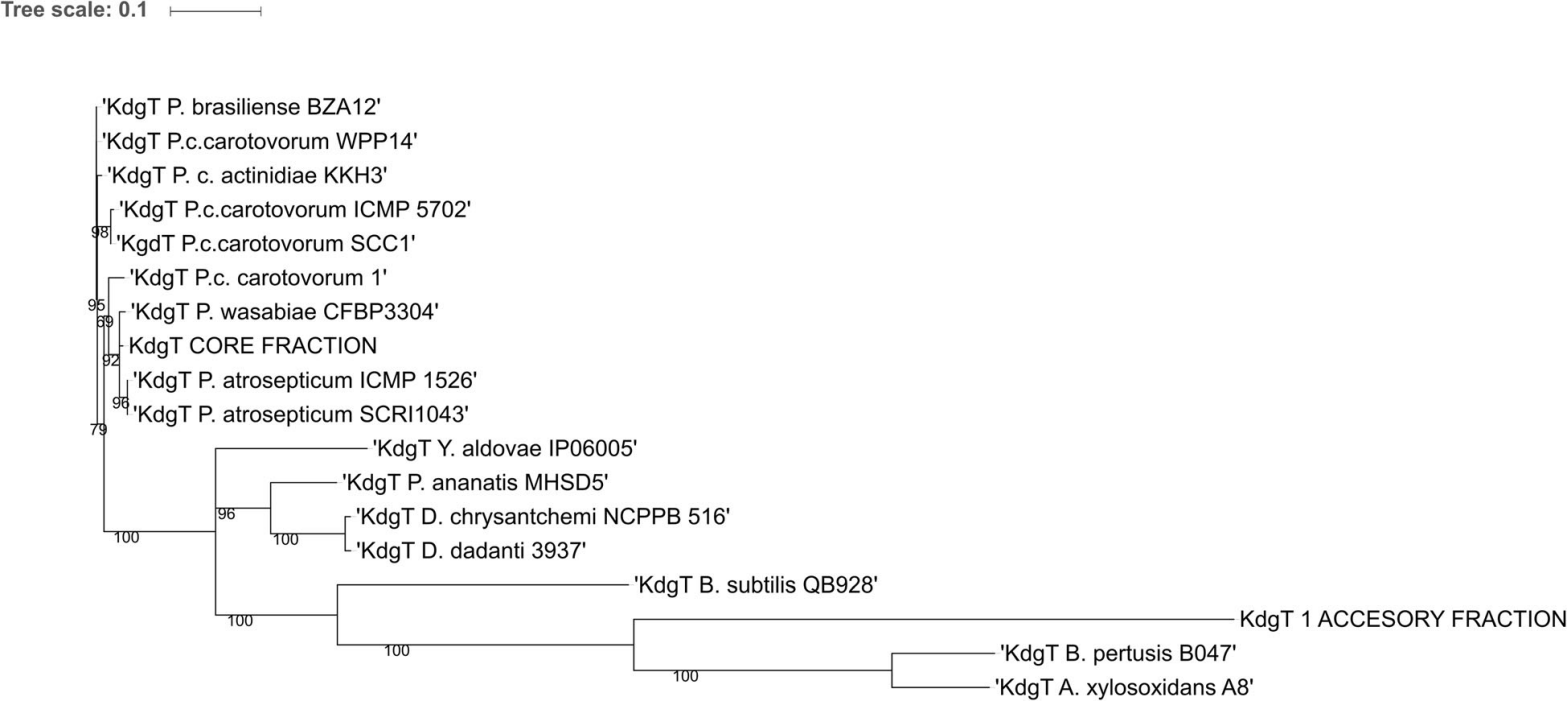
The evolution of KdgT protein among the members of *Pectobacteriaceae* family. The tree was rotted on KdgT of *Dickeya dadantii* 3937 (Dda 3937) and *Dickeya chrysanthemi* NCPPB 516 (Ddh NCPPB 516). Pcc – *P. carotovorum* subsp. *carotovorum,* Pcbr - *P. carotovorum* subsp. *brasiliense,* Pba – *P. atrosepticum,* P. c. actinidiae - *P. carotovorum* subsp. *actinidiae*

Importantly, among the investigated 15 *P. parmentieri* genomes we discovered peculiar differences in the quantities and types of *kdgT* genes encoding a 2-keto-3-deoxygluconate permease and *kdgM* coding for oligogalacturonate-specific porin KdgM [49]. Two different paralogs of *kdgT,* i.e. *kdgT* and *kdgT_1*, were identified. *kdgT* is present in core genomes, while the paralogous copy *kdgT_1* is absent in the genomic sequences of IFB5432, IFB5441, and IFB5623 strains. A dendrogram based on KdgT and KdgT_1 protein sequences, available in GenBank, showed branching out of KdgT_1 far away from the other KdgT orthologs originating from different *Pectobacteriaceae* spp. (Fig. 5). Moreover, KdgT_1 protein is more closely related to KdgT from *Bacillus* spp.

Additionally, three paralogs of the *kgdM* gene were detected among the studied *P. parmentieri* genomes, where *kgdM* encodes oligogalacturonate-specific porin KdgM which expression is strongly induced by pectin derivatives [49]. Precisely, *kdgM_3* is present among all the analyzed genomes, whereas *kdgM*_1 and *kdgM_2* are observed among the analyzed genomes in different combinations. Usually, one strain possesses two paralogs of this gene (except for IFB5408, IFB5427, IFB5626 – these strains only have *kdgM_3*, while IFB5486 and IFB5605 have either *kdgM*_1 or *kdgM_2*). The herein reported gene duplication events might undergo a long-term selection pressure ressulting in P. parmentieri genome plasticity. Similar duplications were observed in the genomes of *Escherichia coli* [29].

All the studied *P. parmentieri* genomes possess genes encoding AHL-dependent quorum sensing system and the components of type 4 and type 6 secretion systems. In contrast, none of the tested genomes showed the presence of genes encoding the type 3 secretion system, which were usually detected in the *Dickeya* and *Pectobacterium* genomes [50]. However, we did notice the presence of genes *fliR* and *flhB* coding for effector proteins of the type 3 secretion system in the core genome fraction. These results are in agreement with some earlier data showing the absence of type 3 secretion system in the genome of *P. parmentieri*, but revealing at the same time the presence of genes encoding its effector proteins [25, 51, 52].

Analysis of the presence of the structural genes within the genomes of *P. parmentieri* strains did not adequately explain differences in the abilities of the tested strains to degrade plant cell wall components. It can be hypothesized that the variation mentioned above might have resulted from differential regulation of expression of virulence-related genes. There have been some indications that regulatory data are more useful for the explanation of phenotypic differences than the pure genomic information [39, 53].

However, all the tested genomes possess regulatory elements affecting the expression of the genes related to virulence: components of AHL-dependent quorum sensing system, 3 functional copies of *kdgR* (gene encoding a global negative regulator of pectin degradation), *crp* (encoding cAMP-activated global regulator), *fur* (coding for ferric iron uptake regulator), *cheZ* (encoding a chemotaxis regulator) and *rcsF* (coding for a stress response system protein) (Additional file 2: Table S1). It can be therefore concluded that future work should be directed towards a better understanding of the regulatory network controlling maceration abilities of different *P. parmentieri* strains.

Unfortunately, we cannot a priori exclude that random drift may be related to the lack of correlation between genomic differences and phenotypic differences, as observed in various host-associated microorganisms [54]; still, the number of sequenced genomes of *P. parmentieri* is too limited to draw a definitive conclusion on such hypothesis.

Furthermore, we have analyzed the genes encoding proteins involved in LPS biosynthesis, motility, chemotaxis, iron uptake and utilization in addition to resistance to oxidative stress (Additional file 2: Table S1). Several genes involved in LPS biosynthesis are present in the accessory pan-genome fraction, which correlated with the observed different degrees of pathogenicity-related traits of the *P. parmentieri* strains. In more detail, superb variation among the strains regarding motility and ability to form biofilm was presented (Table 2)*.* For example, strain IFB5408 showed almost the same ability to form biofilm as *P. aeruginosa* PAO1, and also indicated the most efficient swimming motility (Table 2). Moreover, this strain possesses additional, strain-specific copies of genes encoding lipopolysaccharide ABC transporter substrate-binding protein LptA, LPS export ABC transporter periplasmic protein LptC, and also an additional copy of lipopolysaccharide ABC transporter ATP-binding protein.

The occurrence of two genes of possible eukaryotic origin, for example, plant ferredoxin-like protein and benzoic acid/salicylic acid carboxyl methyltransferase, was confirmed in all the tested genomes.

In the previous studies, *P. parmentieri* SCC3193 was stated to harbour an arsenic resistance cluster [16] common among bacteria that possess it either on the chromosome or on the plasmid. [55]. However, in the core pangenome of *P. parmentieri,* there was only one gene found encoding an arsenate reductase, namely *arsC.* The other members of the arsenic-resistant cluster were enclosed within the unique pan-genome fraction specific to *P. parmentieri* SCC3193 strain (Additional file 2: Table S1). Notably, none of the *arsC* genes present in the *P. parmentieri* genomes analyzed here was located within any MGE as it was the case of *P. parmentieri* SCC3193 genome [16].

### The large mobilome of *P. parmentieri*

A high number of phage-related genes among the analyzed *P. parmentieri* strains was found, constituting especially the accessory and the unique pan-genome fraction. Therefore, a comprehensive evaluation of unique pan-genome fraction content was subsequently performed. *P. parmentieri* SCC3193 strain has the highest number of unique genes, namely 346 genes (Table 3). Besides, six Polish strains possess an intermediate number of unique genes (from 112 to 177).

Regarding unique pan-genome fraction almost half of the genes codes for phage-like proteins (e.g. transposases, phage structural proteins) and several conjugation transfer proteins. As a consequence of these findings, the presence of prophages was evaluated, and up to 4 intact prophages per strain were found (Table 4, Additional file 3: Figure S2). These prophages harboured genes encoding different toxins (e.g. HigB, CcdB, and YafO), bacteriocins, and a few antitoxins genes. In general, the toxins mentioned above are either involved in the inhibition of the gene expression or blockage of the foreign DNA invasion into the bacterial cell.

**Table 4:** Presence of toxins, bacteriocins and antitoxins within phage regions of the analyzed *P. parmentieri* strains

Strain	Intact prophages	Genes encoding toxins in phage regions					Genes encoding antitoxins in phage regions	
		HigB	CcdB	YafO	RelE	Bacteriocins	HigA	YafN
IFB5408	1	–	–	–	–	C5E17_10830	–	–
IFB5427	4	C5E18_11805	–	C5E18_11035	–	–	C5E18_08010	–
IFB5432	2	C5E19_17230	C5E19_17060	–	C5E19_17110	–	–	–
IFB5441	3	–	C5E20_17730	–	–	–	–	–
IFB5485	0	–	–	–	–	–	–	–
IFB5486	3	C5E22_20550	–	–	–	–	–	–
IFB5597	1	–	–	–	–	–	–	–
IFB5604	0	–	–	–	–	–	–	–
IFB5605	4	–	–	C5E24_11080	–	C5E24_10885	C5E24_08305	–
IFB5619	0	–	–	–	–	–	–	–
IFB5623	2	–	–	–	–	–	–	–
IFB5626	3	–	C5E26_17330	–	–	C5E00_05110	C5E00_02270	–
CFBP 8475^T^	1	–	–	–	–	–	A8F97_RS10330	–
SCC3193	0	–	–	–	–	–	–	–
WPP0163	0	–	–	–	–	–	–	–

In more detail, HigB being a ribosome-dependent toxin preferentially cleaving mRNA at adenosine-rich codons is responsible for plasmid stability [56], and have been identified in the genomes of IFB5427, IFB5432 and IFB5486 strains (Table 4). We have also found a putative antitoxin HigA present in prophage regions of the genomes of IFB5427, IFB5605, IFB5626 isolates in addition to CFBP8475^T^. HigB and HigA are present in the genome of IFB5427. However, it is unlikely that they interact with one another because they do not fall under the regulation of the same operon (Additional file 4: Table S2).

The second most common toxin that is known to act on DNA gyrase [57] and is encoded by the F plasmid-carried gene, namely *ccdB*, was found in the genomes of IFB5432, IFB5441 and IFB5623. Besides, a gene coding for YafO toxin, which is responsible for protein synthesis inhibition [58] was detected in the genomes of IFB5427 and IFB5605. Another toxin RelE participating in growth arrest and cell death by inducing mRNA degradation at the ribosomal A-site under stress conditions [59] was encoded by the prophage regions of IFB5432 and IFB5623 sequences. Notably, genes coding for various bacteriocins were identified within the prophage hot spot regions’ of IFB5408, IFB5605 and IFB5626 genomes (Additional file 4: Table S2).

It is worth to underline that only incomplete prophages were spotted in the genomes of IFB5485, IFB5604, IFB5619 and two reference genomes: SCC3193, WPP0163. Accordingly to our findings and as it has been stated before [27] *P. parmentieri* strains are capable of acquiring efficiently large portions of extracellular DNA. Interestingly, some additional copies of the toxins mentioned above were found outside of phage regions; for instance, the *relE* gene is present in each *P. parmentieri* genome in at least a triplicate. Besides, genomes of all the tested strains possess a colicin V coding gene (Additional file 4: Table S2). The latter discovery is especially intriguing as the resulting protein is usually produced from large low-copy plasmids and inhibits translation during amino acid starvation [60]. However, we only confirmed the presence of any plasmid in just one strain, namely *P. parmentieri* IFB5427.

### Shedding light on phage infections

CRISPR-Cas systems provide adaptive immunity in prokaryotes and can be effective against phage infections. They are composed of CRISPR arrays consisting of short repeats separated by spacer sequences derived from invading nucleic acids in addition to the CRISPR-associated (Cas) genes [61]. Four loci of CRISPR arrays (specifically four loci of multiple spacers) were detected in each *P. parmentieri* genome, named here from CRISPR1 to CRISPR4 (Additional file 5: Table S3). After performing clustering based on nucleotide alignment of the extracted sequences, it turned out that almost all the *P. parmentieri* CRISPR are grouping into two separate clades: the first cluster includes two CRISPR repeats: CRISPR1 and CRISPR2 and the second one additional two: CRISPR3 and CRISPR4 (Additional file 6: Figure S3). The genomes of *P. parmentieri* CFBP 8475^T^ (a strain isolated in France) and IFB5486 (strain originating from Belgium) also possess 4 CRISPR repeats. However, their CRISPR sequences are specific and differ from the previously described CRISPR repeats. Notably, CRISPR1 and CRISPR2 group together, similarly to CRISPR3 and CRISPR4 forming also separate clades. We have found differences among quantities of each spacer related to specific CRISPR arrays ranging from 4 spacers up to 94 per one CRISPR array (Additional file 5: Table S3). It was proven before that when a bacterial cell is infected with a new phage, a piece of its foreign DNA can be integrated at the end of the CRISPR array as a new spacer. This spacer can then target and destroy the invading DNA [61]. Therefore, CRISPR regions can reveal information on both phage sequences and order of infection they conducted. However, we cannot be sure if there is always only one new spacer per phage, or if it is possible that more than one spacer is incorporated within the same infection event. Undoubtedly, we can say that there were numerous phage infection events in the analyzed *P. parmentieri* strains.

### *P. parmentieri* plasmid *pPAR01* - a possible source of additional genomic variation

As mentioned before, IFB5427 strain harbours a *pPAR01* plasmid of 101,998 bp with 125 CDS and GC content of 49.45%, which is approximately 1% lower than the GC content of all the analyzed *P. parmentieri* bacterial chromosomes. Among the encoded proteins IncFII (plasmid replication initiator protein), RepA (initiation replication protein essential for plasmid replication), ParM (plasmid segregation), relaxase TraJ (a conjugation system) and a conjugal transfer protein should be listed. Besides, we have found a gene coding for YafN antitoxin located on the *pPAR01* plasmid, and a gene encoding YafO toxin on the chromosome of this strain within a ‘hot spot’, being an entire prophage region. YafO is a toxin which inhibits protein synthesis and constitutes a type II toxin-antitoxin system, in which the YafN acts as an antitoxin. However, we do not possess any evidence that this toxin-antitoxin pair complements each other.

In addition to YafN, *pPAR01* also comprises another putative antitoxin VapB; anyhow, we did not identify a toxin corresponding to this protein either on the plasmid or the chromosome of IFB5427. Besides, *pPAR01* plasmid harbours a toxin RelE, but again without any proper antitoxin gene on any genetic unit.

The here described *pPAR01* plasmid retains other exciting features, which could be related to its possible transmissibility. For instance, anti-restriction proteins ArdA and KlcA, which may allow for the associated MGE to evade the ubiquitous Type I DNA restriction systems in the recipient bacterium [62] resulting in the acquisition of cognate modification. Also, the presence of the gene coding for N6 DNA methylase suggests that maintaining this plasmid may affect histone methylation pattern within the host DNA to avoid further invasion by foreign nucleic acids [63]. *pPAR01* also encodes proteins involved in SOS responses, such as the DNA adenine methyltransferase YhdJ essential for DNA repair, and DNA polymerase V subunit UmuC playing a role in replication under the stress responses [64]. On the other hand, SOS inhibition proteins (PsiA and PsiB), which impede bacterial stress responses are also located on this plasmid. Given reported induction of bacterial SOS responses by conjugative DNA transfer, it can be speculated that the presence of genes coding for SOS inhibition proteins may increase plasmid transferability to the host cell [65].

## Conclusions

In conclusion, the obtained results show differences in the ability of *P. parmentieri* strains to macerate potato tubers tissue in addition to variation in their phenotypic traits. The observed high genomic plasticity can explain these features within *P. parmentieri* species expressed by a high number of MGE found both in the core and particularly in the dispensable pan-genome fraction. The analysis of the genomes of 15 *P. parmentieri* strains indicated that the dispensable pan-genome fraction constitutes 47% of the whole pan-genome. Also, the occurrence of prophage sequences and CRISPR-Cas system elements is abundant in the dispensable genome. The widespread presence of MGE may have caused considerable genome rearrangements in addition to the gene loss, as it was noted in the case of lack of type 3 secretion system components. Moreover, we can hypothesize that such high genomic variation among *P. parmentieri* strains could be the basis for the widespread presence of this species and its potential wide host range. In conclusion, our comparative genomic analysis of *P. parmentieri* highlights the contribution of plastic genomic structure to adaptive lifestyle and ability to survive and cause disease symptoms in different climatic zones.

## Methods

### Strains used in the study

*P. parmentieri* strains used in this study are presented in Table 1. Ten of these strains originating from Poland have been partially described previously regarding their phenotypic features [21]. The additional two strains have been isolated in Belgium by Johan van Vaerenbergh (ILVO, Belgium) and are included in this type of analysis for the first time. Also, we have used *P. parmentieri* CFBP 8475^T^ isolated in France, *P. parmentieri* SCC3193 isolated in Finland, and *P. wasabiae* CFBP 3304 as a closely related species and an outgroup for phylogenetic analyses. For in silico analysis only, *the P. parmentieri* WPP163 isolated in the US was utilized. *Pseudomonas aeruginosa* PAO1 was used as a control in biofilm formation test.

### Phenotypic characterization of *P. parmentieri* strains

*The maceration ability* of the selected *P. parmentieri* strains was evaluated by performing the potato slice assay as previously described [21]. Briefly, potatoes (cv. Lord) were surface sterilized, washed in tap water and cut into slices (approx. 2 cm thick). In each slice, depending on size, two to three holes were pierced and then into each of them 45 μl of bacterial inoculum (10^7^ cfu x ml^− 1^) was placed. Potatoes were incubated for 48 h at 28 °C on moistened linen within closed plastic boxes. Subsequently, maceration spots diameters were measured. The experiment was performed in three biological repetitions each including eight technical ones.

*The activity of pectinases, cellulases, proteases, siderophores* as well as swarming motility was performed as described before [21]. To obtain consistent results and prevent undirected bacterial movement, *swimming motility* was evaluated on NB medium supplemented with 0.3% agar and also 0.4% PGA (Sigma-Aldrich, USA) [66]. *The activity of lipases* was tested on dedicated medium supplemented with Tween 80 and rhodamine B [67], where diameters of the clear halo zones appearing around the colonies were measured. The experiments were performed four times involving two technical replicates in each. Incubations were performed for 24 h at 28 °C.

*Biofilm formation assay* was performed as described by [45] with following modifications: 10 μl of overnight bacterial cultures in LB medium were transferred to Eppendorf tubes with 400 μl of M9 medium supplemented with 0.4% glucose (M9-C). After 16 h of incubation at 18 °C without agitation, 70 μl of 1% (*w*/*v*) crystal violet solution was added to each tube and incubated at room temperature for 20 min. Subsequent washing and OD_565_ measurement were performed as described by [45].

*Statistical significance* of differences noted in the phenotypic assays was analyzed with the use of the *agricolae* package from the R 3.3.1 programming environment [68]. Leven’s test was implemented to verify whether the data variances were equal. Shapiro-Wilk test was used to check whether the data followed a normal distribution. As the requirements of ANOVA were not always fulfilled, Kruskal-Wallis test with Bonferroni correction was applied for multiple comparisons (R *agricolae* package). All statistical hypotheses were tested at *p* < 0.05. For testing the Pearson’s correlation *Hmisc* package with rcorr was utilized.

### Genome sequencing, de novo assembly and annotation

For shotgun genome sequencing, bacterial overnight cultures were grown on LA plates and subsequently sent to GATC Biotech, Konstanz, Germany. DNA was isolated, libraries have been constructed and then subjected to Illumina MiSeq or HiSeq2000 and Pacific BioSciences sequencing methods, to provide both proper coverages, and high-quality reads. Illumina paired-end reads were trimmed with Trimmomatic [69] to remove adapter sequences and poorly sequenced read ends. De novo genome assembly was performed with the use of SPAdes [70]. In the case of 2 genomes (i.e. of IFB5604 and IFB5619 strains), we could not achieve satisfactory assembly to the level of one scaffold. Therefore, MeDuSa: a multi-draft based scaffolder was applied [71] to close scaffolds to a full chromosome on the basis of reference genomes (acquired from NCBI) and also the genomes assembled by us to a single chromosome within this study. Genome annotation was firstly achieved by utilizing Prokka v1.12 [72]. Afterwards, custom-made Python scripts were used to reorient genomes in order to make them start from the *dnaA* gene sequence. Finally, all reoriented genomic fasta outputs were submitted to the NCBI database, and the final annotation was performed by the NCBI Prokaryotic Genome Annotation Pipeline (PGAP, https://www.ncbi.nlm.nih.gov/genome/annotation_prok/). CRISPR spacers were found with the use of CRISPRFinder (http://crispr.i2bc.paris-saclay.fr/Server/).

### Comparative genomic analysis

*Average Nucleotide Identity* (ANI) was calculated for 15 *P. parmentieri* genomes applying ChunLab’s online Average Nucleotide Identity Calculator (EzBioCloud) [73] where *P. parmentieri* CFBP 8475^T^ was used as an internal reference. *Synteny* was evaluated with the use of Mauve [74]. Phast [75] has been implemented for computational identification and visualization of prophages.

*Gene ontology and the pan-genome shape* of *P. parmentieri* strains were established using Roary [76] with the default settings. PGAP annotated sequences were used as an input. Calculations of the Heap’s Law and *construction of the phylogenetic tree* (constructed by hierarchical clustering of gene families established by reciprocal all-against-all protein sequences BLAST) were performed using the *micropan* R package for microbial pan-genomics [34]. *The Class of Genes (COG) annotation* of every protein belonging to the accessory fraction was done with eggNOG 4.5 server [77].

## Additional files

Additional file 1: **Figure S1.** Phylogenetic relatedness of the analyzed *P. parmentieri* strains: A. Genomic Average Nucleotide Identity (gANI) heatmap with dendrograms. B. Gene presence/absence matrix against core pan-genome generated dendrogram. The dendrogram was created basing on hierarchical clustering of the rows. (PNG 1528 kb)

Additional file 2: **Table S1.** Comprehensive analysis with locus_tag information on virulence genes, virulence-related genes, motility, chemotaxis, iron metabolism, resistance to oxidative stress, quorum sensing related genes and regulators within analyzed *P. parmentieri* strains. Format: xlsx file. (XLSX 74 kb)

Additional file 3: **Figure S2.** Synteny of intact prophages within the particular *P. parmentieri* strains. (PNG 1257 kb)

Additional file 4: **Table S2.** Toxins and antitoxins genes encoded within the genomes of analyzed *P. parmentieri* strains. Format: docx. (DOCX 16 kb)

Additional file 5: **Table S3.** The number of CRISPR spacers found per each CRISPR array in the analyzed *P. parmentieri* strains. (DOCX 12 kb)

Additional file 6: **Figure S3.** Clustering of CRISPR arrays from analyzed *P. parmentieri* strains. Two clusters formed from CRISPR1-CRISPR2 and another two CRISPR3-CRISPR4 and two additional clusters formed only from CRISPR arrays from *P. parmentieri* CFBP 8475^T^ and *P. parmentieri* IFB5486. (PNG 1227 kb)

## Abbreviations

ANI: Average nucleotide identity
COG: Class of genes
HGT: Horizontal gene transfer
MGE: Mobile genetic elements
PCWDE: Plant Cell Wall Degrading Enzymes
