# Supplementary figures and images for: High genomic variability in the plant pathogenic bacterium Pectobacterium parmentieri deciphered from de novo assembled complete genomes
Source: BMC Genomics. 2018 Oct 16;19:751. doi: 10.1186/s12864-018-5140-9 (PMC6192338; doi:10.1186/s12864-018-5140-9)

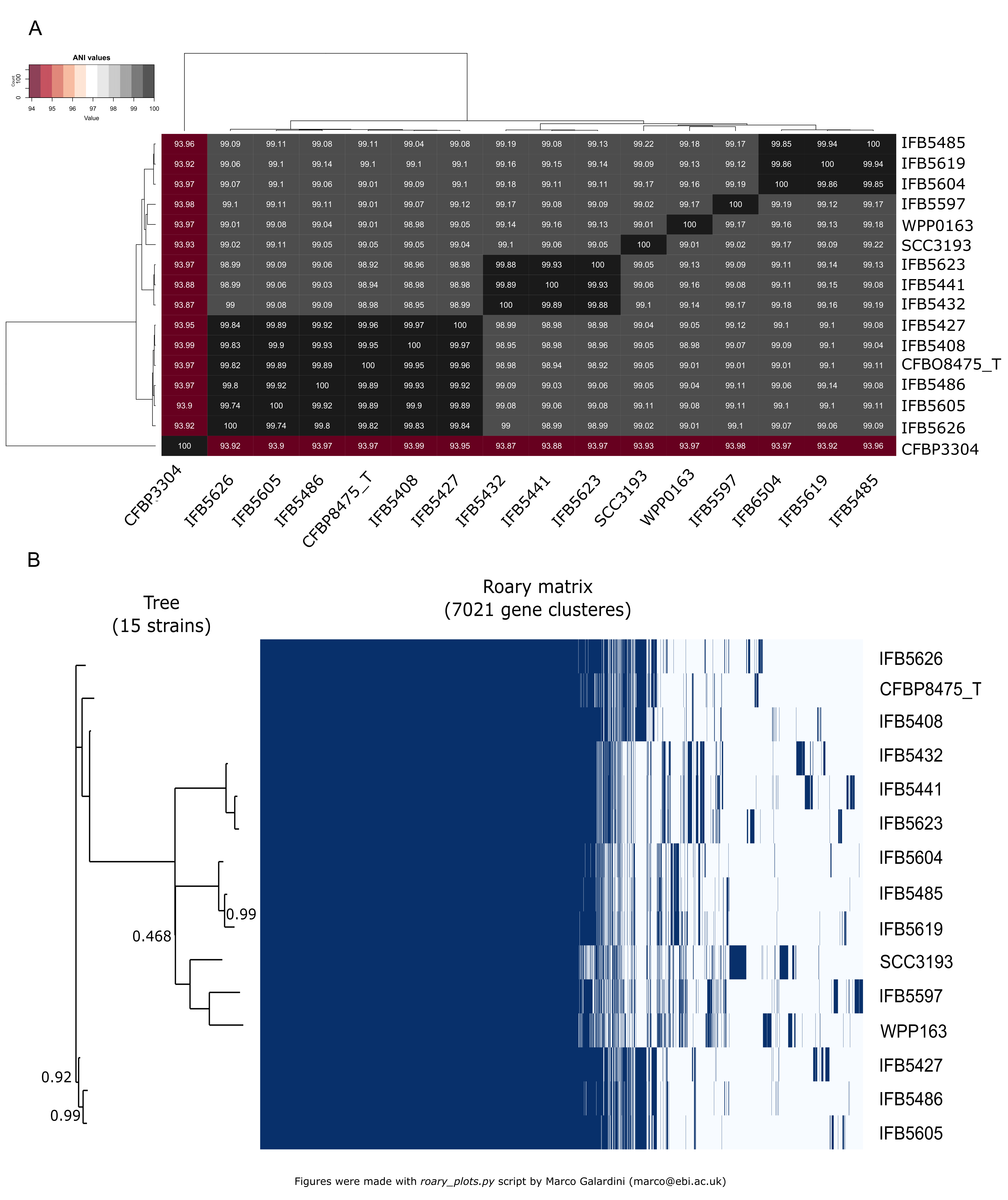

Supplement: Supplementary file 1 — Figure S1. Phylogenetic relatedness of the analyzed P. parmentieri strains: A. Genomic Average Nucleotide Identity (gANI) heatmap with dendrograms. B. Gene presence/absence matrix against core pan-genome generated dendrogram. The dendrogram was created basing on hierarchical clustering of the rows. (PNG 1528 kb) [file 12864_2018_5140_MOESM1_ESM.png]

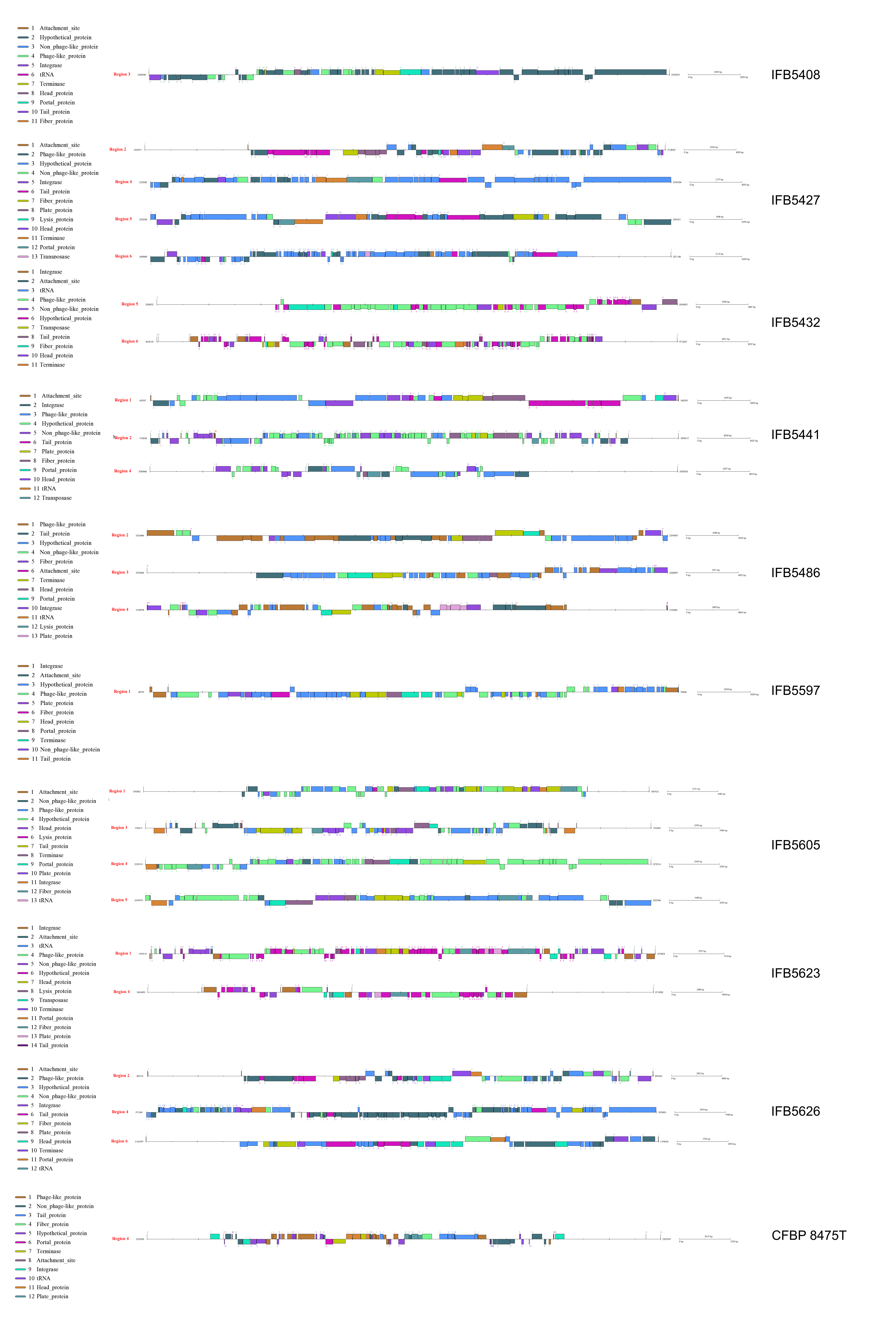

Supplement: Supplementary file 3 — Figure S2. Synteny of intact prophages within the particular P. parmentieri strains. (PNG 1257 kb) [file 12864_2018_5140_MOESM3_ESM.png]

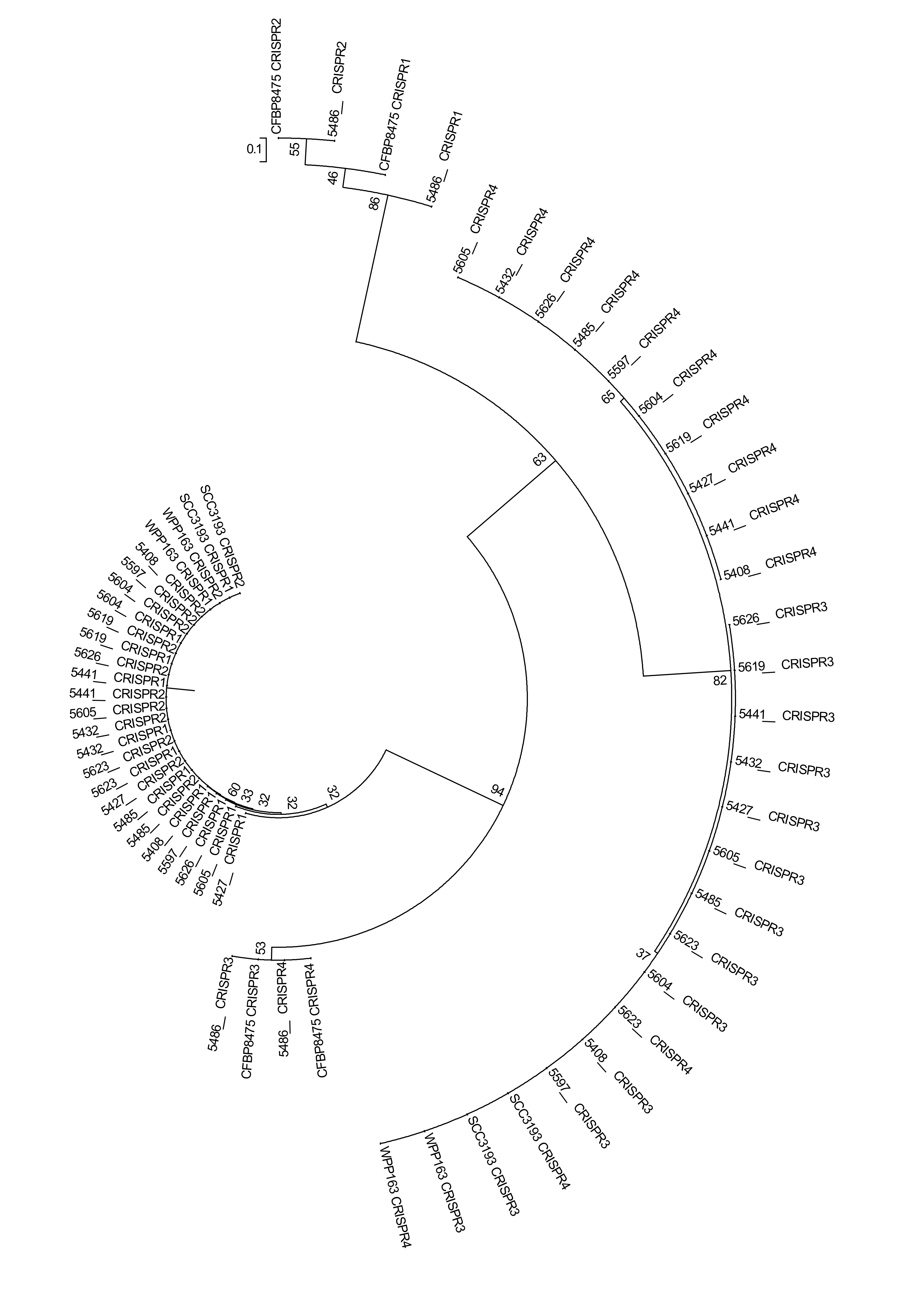

Supplement: Supplementary file 6 — Figure S3. Clustering of CRISPR arrays from analyzed P. parmentieri strains. Two clusters formed from CRISPR1-CRISPR2 and another two CRISPR3-CRISPR4 and two additional clusters formed only from CRISPR arrays from P. parmentieri CFBP 8475T and P. parmentieri IFB5486. (PNG 1227 kb) [file 12864_2018_5140_MOESM6_ESM.png]
