# Supplementary material for: High genomic variability in the plant pathogenic bacterium Pectobacterium parmentieri deciphered from de novo assembled complete genomes
Source: BMC Genomics. 2018 Oct 16;19:751. doi: 10.1186/s12864-018-5140-9 (PMC6192338; doi:10.1186/s12864-018-5140-9)
Supplement: Supplementary file 4 — Table S2. Toxins and antitoxins genes encoded within the genomes of analyzed P. parmentieri strains. Format: docx. (DOCX 16 kb) [file 12864_2018_5140_MOESM4_ESM.docx]

**Supplementary Table 2.** Toxins and antitoxins genes encoded within the genomes of analyzed *P. parmentieri* strains.

|  | Genes encoding toxins | | | | | | Genes encoding antitoxins | |
| --- | --- | --- | --- | --- | --- | --- | --- | --- |
| *P. parmentieri* strains | HigB | CcdB | RelE | YafO | Bacteriocins | Colicins | HigA | YafN |
| IFB5408 | C5E17_02235 | C5E17_17355 | C5E17_00575 C5E17_02230 C5E17_02450 C5E17_02475 C5E17_04455 | - | C5E17_06965 C5E17_10830 | C5E17_07230 | - | - |
| IFB5427 | C5E18_02070 C5E18_11795 | C5E18_18045 | C5E18_00575 C5E18_02065 C5E18_02285 C5E18_02310 C5E18_04290 | C5E18_11025 | C5E18_06800 C5E18_10830 | C5E18_07065 | C5E18_08010 | - |
| IFB5432 | C5E19_02175 | C5E19_17060 | C5E19_00570 C5E19_02170 C5E19_04470 | - | - | C5E19_07125 | - | - |
| IFB5441 | C5E20_02385 | C5E20_08940 C5E20_17730 | C5E20_00575 C5E20_02380 | - | - | C5E20_07355 | - | - |
| IFB5485, GBBC 1786 | C5E21_02410 | - | C5E21_00575 C5E21_02405 C5E21_02725 C5E21_04590 | - | - | C5E21_07365 | - | - |
| IFB5486, GBBC 1809 | C5E22_20550 | C5E22_05160 | C5E22_00575 C5E22_18215 C5E22_20310 C5E22_20335 C5E22_20555 | C5E22_11710 | C5E22_11905 C5E22_15705 | C5E22_15440 | C5E22_14495 | - |
| IFB5597 | C5E04_04005 | - | C5E04_02300 C5E04_04000 C5E04_04230 | - | C5E04_08775 | C5E04_09035 | - | - |
| IFB5604 | C5E23_02415 | C5E23_16370 | C5E23_00575 C5E23_02410 C5E23_02730 C5E23_04595 | - | - | C5E23_07360 | - | - |
| IFB5605 | C5E24_02070 | C5E24_17625 | C5E24_00575 C5E24_02065 C5E24_02285 C5E24_02310 C5E24_04290 | C5E24_11080 | C5E24_07095 C5E24_10885 | C5E24_07360 | C5E24_08305 | - |
| IFB5619 | C5E25_02430 | - | C5E25_00590 C5E25_02425 C5E25_02745 C5E25_04615 | - | - | C5E25_07385 | - | - |
| IFB5623 | C5E26_02180 | C5E26_17330 | C5E26_00575 C5E26_02175 | - | - | C5E26_07105 | - | - |
| IFB5626 | C5E00_20080 | C5E00_11835 | C5E00_18590 C5E00_20075 C5E00_20295 C5E00_20320 C5E00_22475 | - | C5E00_01060 C5E00_05110 | C5E00_01325 | C5E00_02270 | - |
| CFBP 8475^T^ | A8F97_RS16410 | A8F97_RS01525 | A8F97_RS14220 A8F97_RS16205 A8F97_RS16415 A8F97_RS17805 | - | - | A8F97_RS11420 | A8F97_RS10330 | - |
| SCC3193 | W5S_RS02100 | W5S_RS07875 | W5S_RS00575 W5S_RS01670 W5S_RS02095 W5S_RS02295 W5S_RS02600 W5S_RS04100 | - | - | W5S_RS06675 | - | - |
| WPP0163 | PECWA_RS02020 | - | PECWA_RS00575 PECWA_RS01600 PECWA_RS02015 PECWA_RS02475 PECWA_RS04615 | - | - | PECWA_RS07260 | - | - |
