# Supplementary material for: High genomic variability in the plant pathogenic bacterium Pectobacterium parmentieri deciphered from de novo assembled complete genomes
Source: BMC Genomics. 2018 Oct 16;19:751. doi: 10.1186/s12864-018-5140-9 (PMC6192338; doi:10.1186/s12864-018-5140-9)
Supplement: Supplementary file 5 — Table S3. The number of CRISPR spacers found per each CRISPR array in the analyzed P. parmentieri strains. (DOCX 12 kb) [file 12864_2018_5140_MOESM5_ESM.docx]

| Strain | CRISPR1 | CRISPR2 | CRISPR3 | CRISPR4 |
| --- | --- | --- | --- | --- |
| IFB5408 | 17 | 11 | 34 | 26 |
| IFB5427 | 17 | 11 | 34 | 26 |
| IFB5432 | 15 | 4 | 4 | 51 |
| IFB5441 | 15 | 4 | 4 | 52 |
| IFB5485 | 13 | 11 | 17 | 57 |
| IFB5486 | 26 | 34 | 11 | 17 |
| IFB5597 | 14 | 6 | 10 | 69 |
| IFB5604 | 23 | 11 | 26 | 94 |
| IFB5605 | 17 | 11 | 34 | 26 |
| IFB5623 | 13 | 11 | 17 | 57 |
| IFB5626 | 34 | 26 | 17 | 11 |
| CFBP 8475^T^ | 26 | 34 | 13 | 18 |
| SCC3193 | 21 | 13 | 14 | 31 |
| WPP0163 | 16 | 6 | 17 | 25 |

**Supplementary File 3.** The number of CRISPR spacers found per each CRISPR array in the analyzed strains.
